# The Effect of Bacterial AHL on the Cyclic Adenosine Monophosphate Content in Plants According to High-Performance Liquid Chromatography

**DOI:** 10.3390/molecules29051074

**Published:** 2024-02-29

**Authors:** Xuemeng Zhao, Wen Li, Xiliu Li, Zhenhua Jia, Shuishan Song, Qian Zhao

**Affiliations:** 1 School of Biological Science and Engineering, Hebei University of Economics and Business, Shijiazhuang 050061, China; zhaoxuemeng1121@163.com; 2 Biology Institute, Hebei Academy of Sciences, Shijiazhuang 050051, China; 17603297017@163.com (W.L.); 13582194766@163.com (X.L.); zhenhuaj@hotmail.com (Z.J.); shuishans620@163.com (S.S.); 3 Hebei Technology Innovation Center of Microbiological Control on Main Crop Disease, Shijiazhuang 050051, China

**Keywords:** cyclic adenosine monophosphate, plant, high-performance liquid chromatography, *N*-acyl homoserine lactone, determination of content

## Abstract

Cyclic adenosine monophosphate (cAMP) is an important second messenger in cells, mediating various stimulation signals such as the growth and development of organisms and stress and participating in regulating various biological processes of cells. This article explores the quantitative determination of cAMP in plants using High-Performance Liquid Chromatography (HPLC) and applies this method to analyzing the changes in cAMP content during the process of plant response to the bacterial quorum sensing signal *N*-acyl homoserine lactone (AHL). Research has shown that the optimal detection conditions for HPLC are as follows: the chromatographic column is Venusil MP C18 (2), the mobile phase is methanol–water (0.1% trifluoroacetic acid) (*v*:*v*, 10:90), the detection wavelength is 259 nm, the column temperature is 35 °C, and the flow rate is 0.8 mL/min. The precision of the standard sample of this method is 98.21%, the precision of the sample is 98.87%, and the recovery rate is 101.067%. The optimal extraction conditions for cAMP in Arabidopsis are to use 15% methanol ultrasonic extraction for 10 min, followed by a 40 °C water bath for 4 h. Bacterial AHL signal processing can significantly stimulate an increase in cAMP levels in Arabidopsis leaves and roots. The establishment of HPLC detection methods for the cAMP content in plants is of great significance for in-depth research on the signal transduction mechanisms of plant–bacterial interactions.

## 1. Introduction

Cyclic adenosine monophosphate (cAMP) is typically generated from adenosine triphosphate (ATP) through the catalytic action of adenylate cyclase. Its molecular formula is C_10_H_12_N_5_O_6_P, and its hydrate is a white or pale yellow crystalline compound. It is sparingly soluble in water and not easily soluble in ethanol or ether. Additionally, it exhibits strong resistance to acid, alkali, and heat [1]. cAMP is a crucial intracellular second messenger molecule involved in the regulation of various biological processes within the cells. For instance, cAMP can stimulate cell division and proliferation, participate in the regulation of cellular differentiation, and influence the function and development of the nervous system, among other processes [2,3]. Compared to mammals, cAMP generally exhibits lower concentration levels in higher plants. However, it can significantly increase in response to various stimulating factors, thereby inducing signaling effects. The evidence suggests that cAMP is involved in protein kinase-mediated signaling cascades in higher plants. It is also linked to signaling pathways involving calcium/calmodulin, salicylic acid, jasmonic acid, and nitric oxide. Additionally, cAMP interacts with the effects of many hormones, such as abscisic acid, auxins, gibberellins, and others [4]. cAMP signals can have a significant impact on ion transport, the cell cycle, sexual reproduction, temperature and light responses, photosynthesis, aging, and responses to biotic and abiotic stresses in higher plants. As a result, it plays a broad role in the growth, development, and environmental adaptability of plants [5].

*N*-acyl homoserine lactones (AHLs) are a class of compounds produced by Gram-negative bacteria with quorum-sensing effects. These molecules are transmitted and received within bacterial populations through diffusion and perception mechanisms, earning them the designation of inter-bacterial signaling molecules or biosynthetic products [6]. AHL molecules regulate bacterial collective behavior by initiating or inhibiting specific gene expression, including activities such as biofilm formation, biofilm dispersion, fermentation, biodegradation, and biosynthesis [7]. Recent research has confirmed that bacterial AHL molecules can mediate cross-kingdom communication between plants and bacteria. They participate in regulating plant calcium/calmodulin signaling, as well as the transduction of hormone signals such as salicylic acid, jasmonic acid, and auxins. Consequently, they play a crucial role in controlling plant growth, immune response to diseases, and resistance to abiotic stress [8,9,10,11,12,13]. Therefore, the plant signaling pathways and physiological effects induced by AHLs are consistent with the downstream signaling pathways regulated by cAMP. However, whether AHLs can achieve their physiological functions by activating cAMP signaling remains to be explored and demonstrated. To address this question, quantitative detection of the cAMP levels in plant cells becomes particularly important.

Currently, the quantification of cAMP is primarily achieved using methods such as Enzyme-Linked Immunospecific Assay (ELISA), High-Performance Liquid Chromatography (HPLC), mass spectrometry, and fluorescence detection [14,15,16,17,18,19,20]. ELISA relies on the specific antigen–antibody reaction for quantitative analysis, offering high specificity and sensitivity. However, it is constrained by the antibody selectivity, affinity, and susceptibility to interference from other substances [21]. HPLC, on the other hand, separates different components in a sample based on their varying retention times in a stationary phase, providing higher sensitivity and precision compared to ELISA [22]. Mass spectrometry involves the separation of sample molecules using electromagnetic principles, arranging them into spectra based on their mass-to-charge ratios (*m*/*z*), and often requires coupling with HPLC for separation [23]. Fluorescence detection identifies and quantifies substances based on their fluorescence spectra, exhibiting high sensitivity but being influenced by factors such as the selection of fluorescent dyes and sample autofluorescence [24]. Upon comparing these methods regarding sensitivity, specificity, ease of operation, and safety, this study opts for HPLC to quantify cAMP in plant samples. By optimizing parameters like the detection wavelength and mobile phase, the optimal conditions for detection were determined. Six different cAMP extraction methods were screened, and the best extraction method for *Arabidopsis thaliana* was identified. Furthermore, utilizing this optimal method, the study investigated changes in cAMP levels in Arabidopsis leaves and roots before and after treatment with bacterial AHL signaling molecules. The establishment of this HPLC detection method holds significant importance for a thorough exploration of the signal transduction mechanisms underlying the interaction between plants and bacteria mediated by AHL.

## 2. Results and Analysis

### 2.1. Selection of the Chromatographic Conditions

#### 2.1.1. Selection of Detection Wavelength

**Figure 1:**
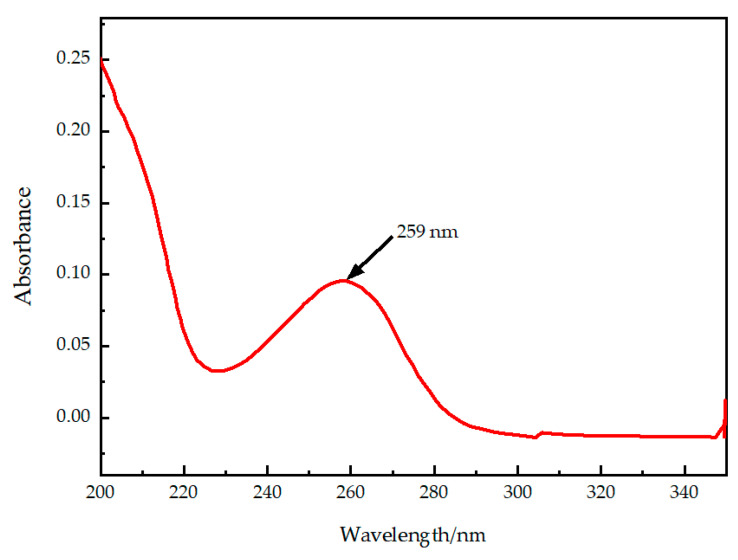
UV full-wavelength scanning result graph of cAMP.

We performed a UV full-wavelength scan on the cAMP standard solution, and the scanning results are shown in Figure 1. cAMP exhibits its maximum absorption peak at a wavelength of 259 nm, so 259 nm is chosen as the detection wavelength.

#### 2.1.2. Selection of the Mobile Phase

**Figure 2:**
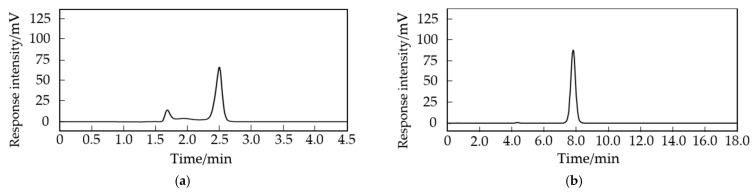
Separation efficiency of different mobile phases. (**a**) Acetonitrile–water mobile phase; (**b**) methanol–water mobile phase.

Two mobile phases, acetonitrile–water (0.1% trifluoroacetic acid) (*v*:*v*, 15:85) and methanol–water (0.1% trifluoroacetic acid) (*v*:*v*, 10:90), were selected for the separation of cAMP, and the results are shown in Figure 2. When acetonitrile–water was used as the mobile phase, the pressure was low, and the peak elution was early, but the separation results contained more impurities. When methanol–water was used as the mobile phase, the pressure was higher, but the substance separation was better, with fewer impurities. Therefore, methanol–water was chosen as the mobile phase.

As a result, the determined conditions for using the HPLC method to measure cAMP are as follows: the chromatographic column is Venusil MP C18 (2), the mobile phase is methanol–water (0.1% trifluoroacetic acid) (*v*:*v*, 10:90), the detection wavelength is 259 nm, the column temperature is 35 °C, and the flow rate is 0.8 mL/min.

#### 2.1.3. Plotting the Standard Curve

**Figure 3:**
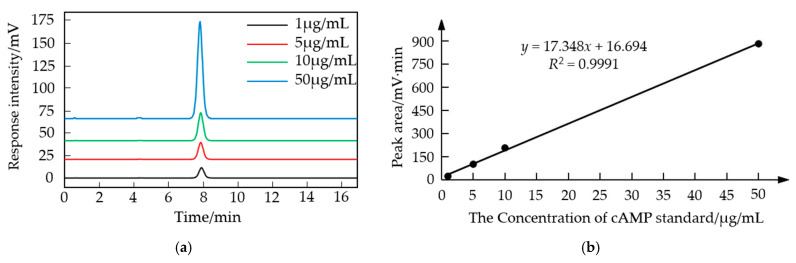
The standard curve of cAMP. (**a**) The chromatograms of cAMP standard samples at different concentrations; (**b**) the standard curve graph.

Using the retention time method for qualitative analysis, the four cAMP standard samples with mass concentrations of 50, 10, 5, and 1 μg/mL were, respectively, detected under the optimal chromatographic conditions, and the results are shown in Figure 3a. The retention times of the cAMP standard samples at different concentrations were consistent, at 7.80 min, and the separation effect of the target peak in the standard samples was good.

We plotted the standard curve with peak area on the y-axis and the concentration of the cAMP standard samples on the *x*-axis, as shown in Figure 3b. The obtained linear regression equation is *y* = 17.348*x* + 16.694, with a correlation coefficient *R*² = 0.9991, which is greater than 0.99, indicating a good linear relationship.

### 2.2. Selection of the Optimal Extraction Method for Arabidopsis cAMP

**Figure 4:**
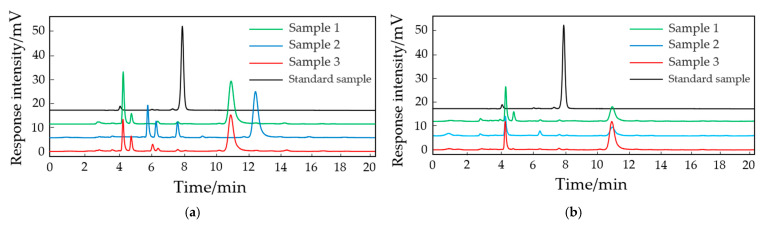

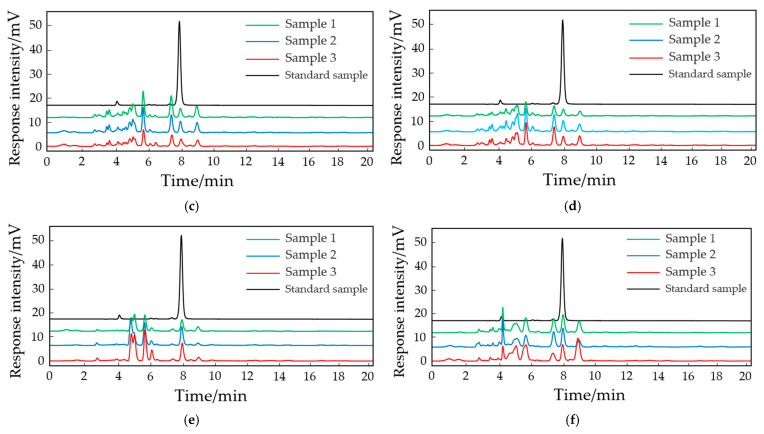
Effect of different extraction methods on cAMP extraction in Arabidopsis leaves. (**a**) Pure water extraction method; (**b**) pure water ultrasonic extraction method; (**c**) pure water boiling extraction method; (**d**) pure water boiling ultrasonic extraction method; (**e**) methanol ultrasonic extraction method; (**f**) acetonitrile ultrasonic extraction method. Explanatory note: Sample 1, Sample 2, and Sample 3 are three repetition samples.

Six different methods, including pure water extraction, pure water ultrasonic extraction, pure water boiling extraction, pure water boiling ultrasonic extraction, methanol ultrasonic extraction, and acetonitrile ultrasonic extraction, were employed to extract cAMP from the Arabidopsis leaves. The detection of cAMP was carried out under liquid-phase conditions using methanol–water (0.1% trifluoroacetic acid) (*v*:*v*, 10:90). The results are shown in Figure 4.

Based on the comparison of the peak times of the standard samples, the pure water extraction sample [Figure 4a] and the pure water ultrasonic extraction sample [Figure 4b] did not show a detectable cAMP peak. The pure water boiling extraction sample [Figure 4c] and the pure water boiling ultrasonic extraction sample [Figure 4d] showed a detectable cAMP peak but with a smaller peak area. The 15% methanol extraction sample [Figure 4e] and the 15% acetonitrile extraction sample [Figure 4f] both exhibited detectable cAMP peaks with larger peak areas. However, the acetonitrile-extracted sample had more impurities. Considering the composition of the mobile phase and the shape and quantity of the sample peaks, the final selected extraction conditions are 15% methanol with ultrasonic disruption for 10 min, followed by a 4 h incubation at 40 °C in a water bath, for extracting cAMP from the plant samples.

### 2.3. Precision Testing

Taking the cAMP standard solution and the extracted solution from the Arabidopsis samples, the aforementioned method was employed for five consecutive injections to assess the precision of the detection method. The results are presented in Table 1. The determination of cAMP in the standard solution ranged from 47.93 to 49.84 μg/mL, with a relative standard deviation (RSD) of 1.79%. For the sample cAMP determination, the results ranged from 2.93 to 3.02 μg/mL, with an RSD of 1.13%, which is below 2%. Hence, this method exhibits good precision.

### 2.4. Recovery Rate Testing

Five Arabidopsis samples with known content were selected, and 0.5 μg of cAMP standard solution was added to each. The content of cAMP was determined using the HPLC method described above, and the recovery rates were calculated. The results are presented in Table 2. The recovery rates for the five samples ranged from 98.741% to 103.072%. When compared to the theoretical recovery rate of 100%, the differences were not statistically significant (*p* > 0.05), indicating no significant discrepancies. The average recovery rate was 101.067% with a relative standard deviation (RSD) of 1.55% (*n* = 5), which is less than 2%. Therefore, the method exhibits good recovery rates.

### 2.5. The Increase in the Plant cAMP Levels Induced by Bacterial AHL

**Figure 5:**
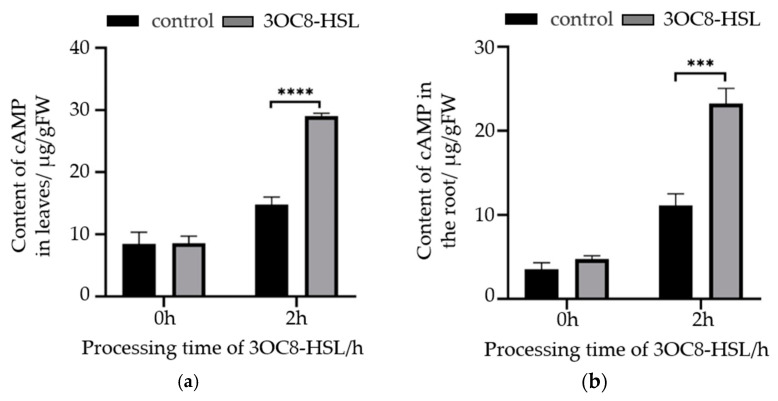
Changes in cAMP level induced by 3OC8-HSL in Arabidopsis. (**a**) Arabidopsis leaf cAMP content; (**b**) Arabidopsis root cAMP content. *** indicates a significant difference with *p* < 0.001, and **** indicates a significant difference with *p* < 0.0001.

Treatment of the Arabidopsis with 10 μmol/L of the bacterial AHL signal molecule 3OC8-HSL was performed. After 2 h, root and leaf tissues were separately collected, and quantitative detection of the cAMP content in the cells was carried out using the above-mentioned method. The results are shown in Figure 5. It is evident that the cAMP content in the Arabidopsis leaves is higher than in the roots, measuring 8.48 μg/gFW and 3.53 μg/gFW, respectively. After 2 h of 3OC8-HSL treatment, the cAMP content in both the leaves and roots of Arabidopsis significantly increased, showing a 96.4% and 108.9% increase, respectively, compared to the untreated control group.

## 3. Discussion

In higher plants, cAMP generally exhibits low concentration levels, and its presence has even been challenging to detect, leading to a long-standing debate about the existence of cAMP in plants [25]. With the rapid development of analytical techniques such as biochemistry, electrophysiology, and molecular biology, it has been confirmed that cAMP is widely present in higher plants and plays a signaling role, exerting significant effects on plant growth, development, and environmental adaptability [26].

Currently, the common methods for detecting cAMP in both animals and plants include ELISA, HPLC and mass spectrometry, and fluorescence detection. Different detection methods have their own advantages and disadvantages. ELISA has high sensitivity, strong specificity, and simple operation but is susceptible to limitations in antibody selectivity and affinity, as well as interference from other substances. HPLC offers higher sensitivity and precision than ELISA, with relatively simple and feasible operation. Mass spectrometry allows for simultaneous qualitative and quantitative detection but often requires coupling with HPLC, and the high cost of mass spectrometers makes it less accessible. Fluorescence detection has the highest sensitivity, but it requires the use of fluorescent dyes and may be affected by sample autofluorescence and self-quenching. Considering factors such as sensitivity, specificity, accuracy, cost, and operational difficulty, this study ultimately chose the HPLC method for the quantitative determination of cAMP in Arabidopsis. The choice of mobile phase is an essential part of chromatographic analysis. Methanol and acetonitrile are ideal mobile phases in high-performance liquid chromatography experiments due to their excellent solubility and volatility [27,28]. Moreover, methanol and acetonitrile can be mixed with water or other solvents to form mobile phases with different compositions, allowing for the adjustment of the mobile phase properties to meet various types of analysis and experimental requirements [29]. In domestic HPLC applications, methanol—0.05 mol/L or 0.1 mol/L potassium dihydrogen phosphate (*v*:*v*, 20:80 or 10:90) is commonly used as the mobile phase for determining the cAMP content in jujube fruits with higher cAMP levels [30,31,32,33]. Other mobile phases such as methanol–acetic acid and acetonitrile–formic acid–ammonium are also employed for determining the cAMP content in plants [34]. Adding inorganic salts to the mobile phase can increase the wear and tear on the chromatographic column. Therefore, we selected salt-free methanol–water and acetonitrile–water as the mobile phases and added 0.1% trifluoroacetic acid as an ion-pairing reagent to improve the peak shape. By comparing the chromatographic peak profiles, we ultimately selected methanol–water (0.1% trifluoroacetic acid) (*v*:*v*, 10:90) as the mobile phase for separating the plant cAMP. The precision of this method reached 98.87%, with a recovery rate of 101.07% and a standard deviation of 1.55%, indicating that the method is accurate and reliable. Additionally, the detection wavelength for cAMP varies in different examples in the literature, with stated values of 254 nm, 256 nm, 259 nm, and 260 nm. Therefore, we performed a full-wavelength scan of cAMP using a UV spectrophotometer and found the maximum absorption peak of cAMP at 259 nm, confirming this wavelength as the detection wavelength for HPLC.

The extraction of bioactive substances from plant organisms involves a myriad of methods, with water immersion, alcohol immersion, and acetonitrile immersion being commonly employed techniques for the extraction of active compounds. In the extraction of cAMP from jujube fruit, the prevalent methods include 80% ethanol extraction or water immersion at different temperatures ranging from 30 to 100 °C. Additionally, ultrasound-assisted extraction and deep eutectic solvent (DES)-ultrasound-assisted extraction have been employed. In a study by Qi et al. [35] analyzing the adenylate cyclase activity of the TIR1/AFB auxin receptors in Arabidopsis, cAMP was extracted using 4% acetic acid and formic acid–ammonium–acetonitrile (5:90). However, this approach was characterized by intricate procedures and yielded a low cAMP content. Given that Arabidopsis is categorized as a plant with low cAMP levels, approximately one-tenth of those in jujube fruit, and considering the properties of cAMP being sparingly soluble in water, poorly soluble in ethanol or ether, and exhibiting strong resistance to acids, alkalis, and heat, we selected six distinct extraction methods. These methods included pure water extraction, pure water ultrasound-assisted extraction, pure water boiling extraction, pure water boiling ultrasound-assisted extraction, methanol ultrasound-assisted extraction, and acetonitrile ultrasound-assisted extraction. Comparing the peak times with the cAMP standard, it was observed that pure water extraction and pure water ultrasound-assisted extraction did not produce cAMP peaks, indicating that these two methods were insufficient for extracting an adequate amount of cAMP. Moreover, the peak areas for cAMP in pure water boiling extraction and pure water boiling ultrasound-assisted extraction were smaller than those in the methanol and acetonitrile extractions, suggesting a lower extraction efficiency. Although the peak areas for cAMP in the methanol and acetonitrile extractions were similar, acetonitrile extraction exhibited more sample impurities. Consequently, the optimal extraction method for Arabidopsis cAMP was determined to be methanol ultrasound-assisted extraction.

In animal tissues, the cAMP level typically ranges from 100 to 500 nmol/L, while in many plants, it is generally below 20 nmol/L. Plants maintain their endogenous cAMP at a low steady state, facilitating a rapid increase under stimulating conditions for signaling purposes. Factors such as red light, far-red light, mechanical damage, heat stress, Ca^2+^, auxins, and pathogen stimuli can significantly influence the cAMP levels in plants [36,37,38,39]. The bacterial quorum-sensing signal molecule AHL mediates inter-kingdom communication between plants and bacteria, activating plant Ca^2+^ signals and ROS signals, as well as hormone signals such as SA, JA, and IAA, thereby influencing plant growth, development, and responses to stress. Genetics and pharmacological assays showed that G protein α subunits participate in the promotion of Arabidopsis primary root elongation as induced by AHL [40]. The luminometric method and electrophysiological approaches showed that the Ca^2+^ level was significantly increased with the addition of AHL and the Ca^2+^ contributing to the increase in [Ca^2+^]cyt was mobilized from the extracellular medium via the plasma membrane Ca^2+^ channels but not from the intracellular Ca^2+^ stores [41]. The downstream G protein pathway primarily involves the activation of adenylate cyclase, a downstream effector, which catalyzes the conversion of ATP into cAMP. This process further activates the calcium channels in the plasma membrane, ultimately regulating the influx of extracellular Ca^2+^ and downstream cellular effects [42]. In the study of the transduction network of plant-induced bacterial AHL signals, the AHL-induced plant cAMP signal serves as a crucial node. Using HPLC analysis, we successfully examined the changes in the cAMP levels in Arabidopsis roots and leaves before and after treatment with 3OC8-HSL. We found that 3OC8-HSL can rapidly elevate the levels of Arabidopsis cAMP by more than two times within a short period (2 h), indicating that 3OC8-HSL can activate the cAMP signal.

Studying the role of cAMP in plant cells responding to bacterial AHL signals contributes to a deeper understanding of the interaction mechanisms between plants and bacteria. By investigating the changes in cAMP and its correlation with bacterial signals, we can uncover the immune response mechanisms of plants to bacterial infections and pave the way for new strategies to enhance plants’ disease resistance and stress tolerance. Additionally, delving into the regulatory role of cAMP in plants also helps us expand our understanding of the cellular signaling networks in plants, laying the groundwork for further research into plant growth, development, and adaptability.

## 4. Materials and Methods

### 4.1. Materials

#### 4.1.1. Samples and Reagents

Wild-type *Arabidopsis thaliana* Col-0 (maintained in our laboratory); *N*-(3-oxooctanoyl)-l-homoserine lactone (3OC8-HSL, a type of AHL molecule, from Sigma-Aldrich Company, St. Louis, MO, USA); cAMP standard (from Sigma Company, Ronkonkoma, NY, USA); methanol (chromatography-grade, from Thermo Fisher Scientific, Waltham, MA, USA); trifluoroacetic acid (chromatography-grade, from Aladdin Reagent Co., Ltd., Shanghai, China); acetonitrile (chromatography-grade, from Anhui Tiandi High Pure Solvent Co., Ltd., Anqing, China).

#### 4.1.2. Experimental Instruments

High-performance liquid chromatography system LC-20A (Shimadzu Corporation, Kyoto, Japan); chromatography column Venusil MP C18 (2) (4.6 mm × 250 mm × 5 μm) (Agilent Technologies, Santa Clara, CA, USA); metal bath H2O3-100C (Coyote Bioscience Company, Beijing, China); ultrasonic cell disruptor (Ningbo Xinzhi Biotechnology Co., Ltd., Ningbo, China); UV-visible spectrophotometer UV-2, 600 (Ningbo Shimadzu Vacuum Technology Development Co., Ltd., Ningbo, China).

### 4.2. Methods

#### 4.2.1. Preparation of cAMP Standard

Dissolve the cAMP standard in ultrapure water to prepare a stock solution of 100 μg/mL. Then, dilute this stock solution stepwise with ultrapure water to obtain cAMP standard solutions with concentrations of 50, 10, 5, and 1 μg/mL.

#### 4.2.2. Selection of Chromatographic Conditions

Perform UV full-wavelength scanning on the cAMP standard solution and determine the wavelength corresponding to the maximum absorption peak as the detection wavelength for HPLC. Next, compare the use of two different mobile phases, acetonitrile–water (0.1% trifluoroacetic acid) (*v*:*v*, 15:85) and methanol–water (0.1% trifluoroacetic acid) (*v*:*v*, 10:90), for the separation of the samples using a reverse-phase C18 chromatography column. Maintain the column temperature at 35 °C and a flow rate of 0.8 mL/min and inject 10 μL of the sample for analysis.

#### 4.2.3. Standard Curve Plotting

Using methanol–water (0.1% trifluoroacetic acid) (*v*:*v*, 10:90) as the mobile phase, detect a series of concentrations (50, 10, 5, and 1 μg/mL) of cAMP standard solution. Record the chromatographic peak profiles, plot a standard curve with cAMP concentration on the x-axis and cAMP peak area on the y-axis, and obtain the linear regression equation.

#### 4.2.4. Arabidopsis Thaliana Cultivation

The seeds of the *Arabidopsis thaliana* wild type Col-0 were sterilized with 75% ethanol for 30 s, followed by triple washing with sterile water. Subsequently, they were immersed in 25% sodium hypochlorite for 5 min, followed by another five washes with sterile water. The seeds were then clustered on MS agar plates and vertically cultured at 22 °C in a growth chamber with a 16 h light/8 h dark cycle for 10 days. Afterward, they were transferred into Hoagland nutrient solution and cultured for an additional 12 days for cAMP extraction.

#### 4.2.5. Preparation and Treatment of the AHL Molecule Solutions

The 3OC8-HSL (*N*-(3-oxooctanoyl)-homoserine lactone) drug powder was dissolved in ultrapure water to prepare a stock solution with a concentration of 10 mmol/L. The solution was then filtered through a 0.22 μM membrane and aliquoted for future use. The *Arabidopsis thaliana* wild type Col-0 was cultured in Hoagland nutrient solution for 12 days. Subsequently, a solution of 3OC8-HSL with a final concentration of 10 μmol/L was added. As a control, Arabidopsis plants without the addition of 3OC8-HSL were simultaneously cultivated in hydroponic conditions. The leaf and root samples were collected at both 0 h and 2 h after treatment for the extraction of cAMP.

#### 4.2.6. Extraction of cAMP

Approximately 0.2 g of Arabidopsis leaves or roots was taken and ground into powder with liquid nitrogen. The extraction solvent was added in a 1:5 ratio, and six methods were employed for cAMP extraction: pure water extraction, pure water ultrasonic extraction, pure water boiling extraction, pure water boiling ultrasonic extraction, methanol ultrasonic extraction, and acetonitrile ultrasonic extraction. The extraction process was conducted under the conditions of immersion in a 40 °C water bath for 4 h.

#### 4.2.7. Precision Experiment

Solutions of the standard substances at a concentration of 50 μg/mL and the samples at a concentration of 3 μg/mL were filtered through a 0.22 μM membrane. The liquid samples were then injected into the liquid chromatograph five times under the selected optimal chromatographic conditions. The peak area of cAMP in the samples was determined, and the cAMP concentrations and the relative standard deviation (RSD) values were calculated based on the standard curve for the five replicates.

#### 4.2.8. Sample Recovery Rate Experiment

Five aliquots of the previously measured sample solutions, each containing 0.2 mL, were taken. To each aliquot, 0.1 mL of a standard solution containing cAMP at a concentration of 5 μg/mL was added, and it was mixed thoroughly and filtered through a 0.22 μM membrane. The cAMP content was determined using the selected optimal chromatographic conditions. The recovery rate of the added samples was calculated, and the relative standard deviation (RSD) was computed. The sample recovery rate can be calculated using the following formula.

Recovery Rate = [m_0_/(m_1_ + m_2_)] × 100%, (1)

In the above equation, (m_0_) represents the measured amount of cAMP in micrograms (μg); (m_1_) represents the added amount of cAMP in micrograms (μg); and (m_2_) represents the cAMP content in the sample in micrograms (μg).

#### 4.2.9. Statistical Analysis

The experimental data were analyzed using GraphPad Prism statistical software 8.0.2, and significance analysis was conducted using the *t*-test (*** indicates a significant difference with *p* < 0.001, **** indicates a significant difference with *p* < 0.0001).

## 5. Conclusions

A method for the quantitative determination of cAMP content using HPLC was established by selecting the detection wavelength and mobile phase. A reverse C18 chromatographic column was employed, with 10% methanol–water (0.1% trifluoroacetic acid) as the mobile phase. The cAMP chromatographic peak was detected at a wavelength of 259 nm, and cAMP was quantified using a standard curve. The method’s sensitivity, precision, and accuracy were validated using precision and recovery experiments. The extraction conditions for cAMP in *Arabidopsis thaliana* samples were screened, and the optimal extraction conditions were determined to be 15% methanol ultrasonic extraction for 10 min, followed by a 4 h water bath at 40 °C. The application of the above method was used to analyze the changes in the cAMP levels in Arabidopsis before and after treatment with the bacterial AHL molecule 3OC8-HSL. It was found that 3OC8-HSL could significantly induce an increase in cAMP levels in the roots and leaves of Arabidopsis, laying the methodological foundation for further analysis of the signal transduction mechanisms of cAMP in AHL-mediated plant–bacteria interactions.

## Figures and Tables

**Table 1:** Precision of HPLC method for determining cAMP.

Serial Number	Standard Determination		Sample Determination	
	cAMP Content/μg/mL	Relative Standard Deviation/%	cAMP Content/μg/mL	Relative Standard Deviation/%
1	49.83773	1.791772	2.974983	1.133876
2	49.68457		3.021732	
3	49.64117		2.977173	
4	47.93290		2.933018	
5	48.35555		3.004900	

**Table 2:** Arabidopsis spiked recovery rate.

Serial Number	cAMP Content in the Sample/μg	Amount of cAMP Added/μg	HPLC Results/μg/mL	Rate of Recovery/%	Average Recovery Rate/%	Relative Standard Deviation/%
1	0.926504	0.5	4.825110	101.4742	101.067	1.553644
2	0.777231		4.388229	103.0721		
3	0.612843		3.662785	98.7413		
4	0.570371		3.591480	100.6608		
5	1.253136		5.924833	101.3869		

## Data Availability

This article is primarily focused on establishing a quantitative measurement technique for plant cAMP. Our main exploration centered around the extraction of plant cAMP and the utilization of HPLC for quantitatively detecting the cAMP levels. Through this exploration, we identified a more effective methodology, which was subsequently preliminarily applied. Specifically, this method was used to analyze the variation in the cAMP levels in plant cells responding to bacterial AHL signals. This sets the groundwork for further research into the AHL-mediated mechanism of plant–bacteria interactions. The entire text includes some crucial data from the experimental process to support the article’s arguments, allowing for replication and experimental validation. However, due to the requirements of subsequent experiments, there are still some data points that have not been disclosed. These undisclosed data will be made public once all experiments are completed. Nevertheless, the absence of these undisclosed data does not hinder the comprehension and validation of the article. Hence, we have made the above statement.

## Abbreviations

AHL: *N*-acyl homoserine lactone; 3OC8-HSL: *N*-3-oxo-octanoyl homoserine lactone; cAMP: cyclic adenosine monophosphate; HPLC: High-Performance Liquid Chromatography; ELISA: Enzyme-Linked Immunospecific Assay; RSD: relative standard deviation; UV: ultraviolet; DES: deep eutectic solvent; TIR1/AFB: Transport Inhibitor Response 1/Auxin-Signaling F-Box; ROS: reactive oxygen species; SA: salicylic acid; JA: jasmonic acid; IAA: Indole-3-Acetic Acid.
